# Correction: Pentraxin 3 in the cerebrospinal fluid during central nervous system infections: A retrospective cohort study

**DOI:** 10.1371/journal.pone.0332720

**Published:** 2025-09-17

**Authors:** Martin Munthe Thomsen, Lea Munthe-Fog, Pelle Trier Petersen, Thore Hillig, Lennart Jan Friis-Hansen, Casper Roed, Zitta Barrella Harboe, Christian Thomas Brandt

In Fig 2, the ROC curve panel D is missing the text “100% - Specificity%”. Please see the correct Fig 2 here.

**Fig 2:**
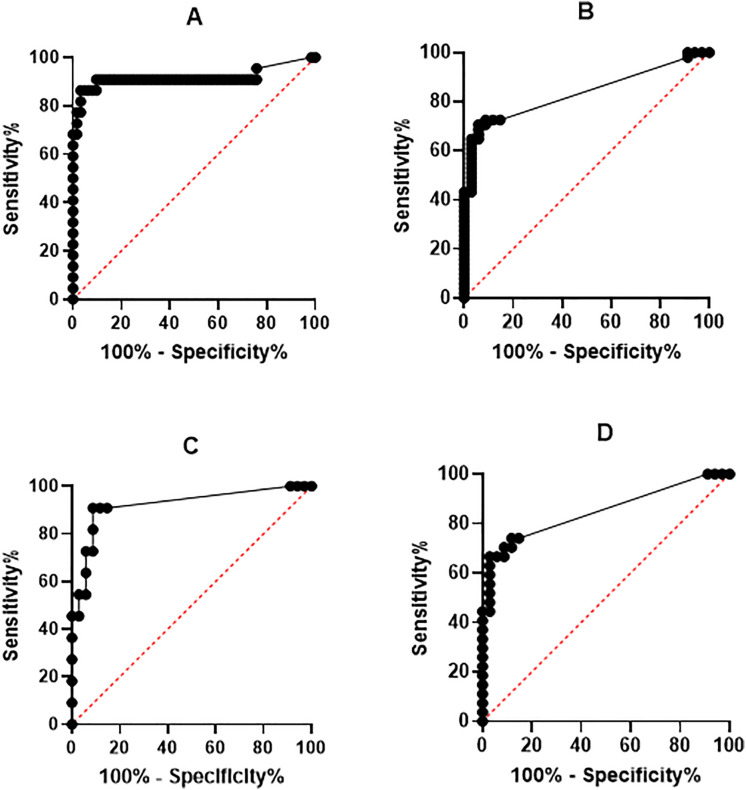
ROC curves for PTX3 diagnostic abilities.

A) Bacterial meningitis versus Viral meningitis and Viral encephalitis. B) Viral meningitis versus Control group. C) Viral encephalitis versus Control group. D) Lyme disease versus Control group.

In Fig 3, a dot plot for CSF cell count is missing. Additionally, the dot plot for CSF polynuclear cells is shown twice, overlapping another dot plot. Please see the correct Fig 3 here.

**Fig 3:**
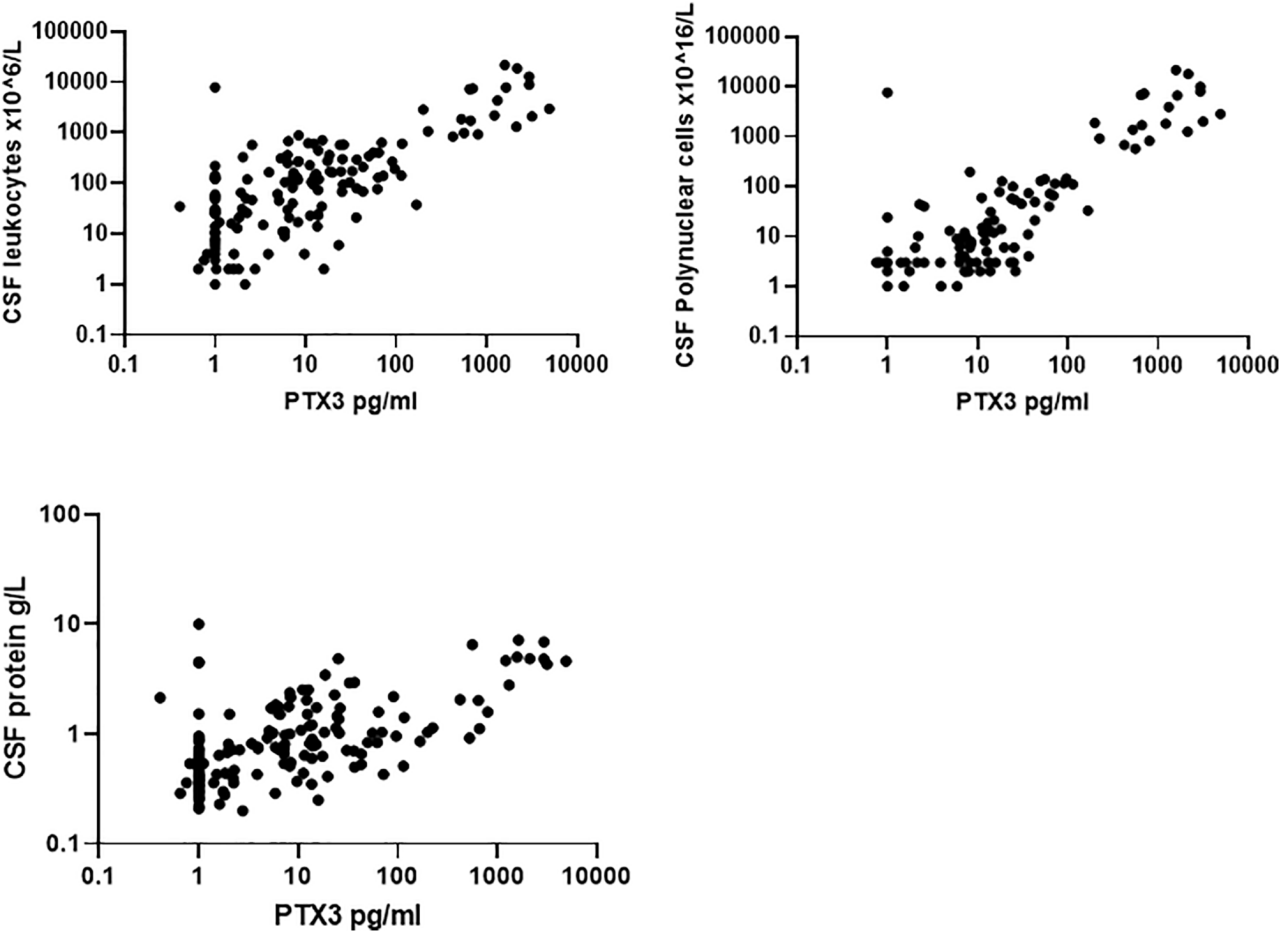
PTX3 correlation to CSF cell count, CSF polynuclear cell count and CSF protein concentration.
